# The value of myocardial work in patients with left ventricular hypertrophy

**DOI:** 10.1007/s10554-023-02818-w

**Published:** 2023-03-16

**Authors:** Jiali Fan, Changsheng Ma, Heng Wang, Bingyuan Zhou

**Affiliations:** The First Affiliated Hospital of Soochow University, Suzhou, China

**Keywords:** Myocardial work, Left ventricular hypertrophy, Fabry disease, Cardiac amyloidosis, Hypertension

## Abstract

Myocardial work derived from pressure-strain analysis resembles a novel non-invasive method for myocardial function evaluation. Left ventricular hypertrophy (LVH) is commonly detected in Fabry disease (FD), cardiac amyloidosis (CA) and hypertension (HTN). The study aimed to demonstrate the characteristics of myocardial work in patients with LVH suffering from FD, CA, and HTN. Echocardiography were performed in patients with LVH suffering from FD (n = 13), light chain associated cardiac amyloidosis (AL-CA) (n = 29) and HTN (n = 72), 25 healthy controls were also included in the current study. Conventional and myocardial work parameters were assessed and compared among FD, AL-CA, HTN and controls. Patients with FD and AL-CA were included in the group of infiltrative cardiomyopathy. Logistic regression analysis was used to identify independent predictors for discriminating infiltrative cardiomyopathy from hypertension. Compared with controls, HTN patients had preserved global work index (GWI) and global constructive work (GCW) and reduced global longitudinal strain (GLS) and global work efficiency (GWE) (p < 0.05), but patients with FD and AL-CA had reduced GLS, GWI, GCW and GWE and increased global wasted work (GWW) (p < 0.05). GWI and GCW could discriminate infiltrative cardiomyopathy from HTN independently with high accuracy (GWI cut-off value 1626 mmHg%, sensitivity 0.87, specificity 0.82, area under the curve 0.90; GCW cut-off value 2021 mmHg%, sensitivity 0.84, specificity 0.88, area under the curve 0.91). GWI and GCW were reduced in FD and AL-CA patients, but not in patients with HTN. Myocardial work had an addictive value in differentiating infiltrative cardiomyopathy from hypertension.

**Supplementary Information:**

The online version contains supplementary material available at 10.1007/s10554-023-02818-w.

## Introduction

Left ventricular (LV) hypertrophy (LVH) is a frequent imaging finding encountered in clinical practice, but it remains challenging to determine the etiology of left ventricular hypertrophy. LVH is commonly detected in cardiac amyloidosis (CA), Fabry disease (FD), and hypertension (HTN). Light chain associated CA (AL-CA) is characterized by progressive deposition of extracellular immunoglobulin-derived light chains [1]. Fabry disease is an X-linked lysosomal storage disorder caused by deficient enzyme activity of -galactosidase A (-Gal A) that leads to the intracellular deposition of complex glycosphingolipids [2–4]. Both FD and CA are infiltrative cardiomyopathies present with thickened ventricular walls. Besides infiltrative cardiomyopathy, LVH is also common in hypertension disease, which is an adaptation response to hemodynamic overload [5]. The pathophysiologic mechanisms responsible for hypertrophy and treatment strategies of the above diseases are not the same, so identification of the etiology of LVH is crucial for disease management.

LV global longitudinal strain (GLS) measured by speckle tracking echocardiography has been reported benefit for detecting systolic dysfunction in patients with CA, FD and HTN [5–7], which is more sensitive than left ventricular ejection fraction (LVEF), but it is influenced by loading conditions. Myocardial work is a novel non-invasive method to characterize myocardial deformation in relation to afterload conditions [8]. Previous studies demonstrated that myocardial work indices were impaired and had prognostic value in patients with CA and FD [9, 10], and it has also been proved that global work index (GWI) and global constructive work (GCW) were increased in patients with hypertension [11, 12]. However, there is a paucity of studies comparing myocardial work parameters in patients with FD, CA, and hypertension. In the present study we aimed to describe the characteristics of myocardial work in patients with LVH suffering from FD, AL-CA, and hypertension.

## Methods

### Population

Study approval was obtained from the institutional enrolling board of the first affiliated hospital of Soochow university and all participants provided written consent. From January 2018 to November 2021, Patients with LVH experiencing FD (n = 13), AL-CA (n = 29) and HTN (n = 72) were prospectively recruited, 25 healthy controls were also included in the current study. None of the 13 FD patients had started enzyme replacement therapy at the time of enrolment. Genotype and clinical characteristics for FD patients were provided in Supplementary material Table S1. AL-CA patients and FD patients were included in infiltrative cardiomyopathy group. LVH was defined as a septal or posterior wall thickness > 11 mm and LV mass > 102 g/m^2^ for man and LV mass > 88 g/m^2^ for woman [13]. Patients with moderate or greater valvular heart disease, coronary artery disease and poor image quality were excluded.

### Definition of Fabry disease, light-chain associated cardiac amyloidosis, and hypertension

The diagnosis of Fabry disease was confirmed by mutation analysis genetic testing and/or reduced α-galactosidase A activity in peripheral blood lymphocytes (male subjects). Cardiac AL amyloidosis was diagnosed by combination of typical features on echocardiography and histologically proven systemic AL amyloidosis. Diagnosis of systemic AL amyloidosis was defined by peripheral tissue biopsy demonstrating Congo red-positive deposits with typical birefringence under polarized light together with unequivocal staining for kappa or lambda light chain by immunofluorescence or immunohistochemistry. Cardiac involvement on echocardiography included: increased LV wall thickness (in the absence of any other plausible causes of LV hypertrophy), granular sparkling appearance of the myocardium, increased thickness of atrioventricular valves, right ventricular free wall, or interatrial septum, and pericardial effusion [14, 15]. Hypertension was defined by a repeatedly measured systolic blood pressure ≥ 140 mmHg or diastolic blood pressure ≥ 90 mmHg or if the subject was receiving optimal antihypertensive pharmacotherapy based on the current guideline [16].

### Echocardiography

We used a commercially available ultrasound system (Vivid E95, GE Healthcare Horten, Norway) with a 3.5-MHz-phased array transducer (M5S). Assessment of two-dimensional echocardiographic imaging in all subjects was performed in accordance with current guidelines [17]. LV septal and posterior wall thicknesses were measured, and relative wall thickness (RWT) was calculated [17]. Doppler measurements included mitral inflow early diastolic wave (E) and late diastolic (A) wave. Tissue Doppler early peak diastolic wave (e′) was obtained from the apical four-chamber view at the basal level of the septum as well as the lateral wall, thereafter E/e′ was calculated as mitral E wave divided by the average of septal and lateral e′. LV end-diastolic volume (LVEDV) and LVEF were calculated using Simpson’s biplane method. Measurement for left atrial volume (LAV) was based on area-length technique on apical four- and two-chamber views. LVEDV index (LVEDVi) and LAV index (LAVi) were calculated by dividing LVEDV and LAV by body surface area (BSA) respectively. Two-dimensional left ventricular GLS was analyzed using automated function imaging in standard 2D cine loops with a frame rate > 55 frames/s. Left ventricular GLS was calculated using a 17-segment model at the time in systole when the value peaked. The principles for estimation of LV pressure and work have previously been described [8]. In this method, we used a previously generated empiric reference curve for LV pressure assessment. This reference curve is individualized by scaling the amplitude using measured systolic cuff pressure. Subsequently, a pressure–strain curve is obtained by fitting the individualized reference curve in time according to aorta and mitral valve opening and closing. Myocardial work indices that were calculated were GWI, GCW, global wasted work (GWW) and global work efficiency (GWE). We calculated the average of apical, midventricular, and basal segments for both GLS and GWI. Apical-to-basal ratio (ABr) of GLS and GWI were calculated by dividing the average of apical segments by the average of the basal segments. Relative apical sparing pattern (RASp) of GLS and GWI were calculated by dividing the average apical segments by the average of the basal and midventricular segments [18]. Data were analyzed offline using dedicated software (EchoPAC PC version 203, GE Vingmed Ultrasound AS, Horten Norway).

### Statistic

Analyses were performed using SPSS version 25.0. Continuous variables which were normally distributed were presented as mean ± SD. Variables that were not normally distributed were presented as median with inter-quartile ranges (IQR 25–75th percentile). Categorical variables were expressed as absolute numbers and respective percentages. Differences of continuous data among 4 groups were compared using 1- or 2-way ANOVA after normalization if indicated. Appropriate post hoc tests were used for multiple comparisons (Tukey if equal variances assumed; Games-Howell if equal variances not assumed). Nonnormally distributed variables were normalized before analysis using natural logarithm or inverted values. Differences in categorical variables among groups were compared using χ^2^ test for overall test, and Fisher’s exact test for pairwise group test. Bonferroni-corrected p values were calculated for multiple comparisons. FD and AL-CA were included in infiltrative cardiomyopathy group, which was compared with HTN group. For comparison between two groups, continuous variables were compared by T test and Mann–Whitney U test, as appropriate. Categorical variables were compared by χ^2^-test. A difference was considered significant when the p value was < 0.05.

Univariate logistic regression analysis was used to find predictors for discriminating infiltrative cardiomyopathy from HTN. Multivariate logistic regression analysis was performed by entering the model a set of variables that were considered significant on univariate analysis (p < 0.05) to identify independent predictors for discriminating infiltrative cardiomyopathy from HTN. Variance inflation factor (VIF) was used to quantify the potential multicollinearity among covariates in the model. As a common rule of thumb, a VIF > 5 was considered for the presence of multicollinearity. In the current study, GLS, GWI, GCW, GWW, GWE and systolic blood pressure had multicollinearity, so they were analyzed separately in multivariate models. Receiver operating characteristic (ROC) curves were used to assess diagnostic accuracy for discriminating infiltrative cardiomyopathy from hypertension. Area under the curve (AUC), sensitivity and specificity were calculated from the true/false, positive/negative classifications using standard definitions. Bland-Altman [19] analysis was performed in 20 randomly selected patients to measure inter-observer and intra-observer variability.

## Results

There was no significant difference in gender distribution among the four groups. All the three groups with LVH had significantly increased left ventricular mass index (LVMI), LAVi and E/e′ than healthy controls. Patients suffering from HTN had larger BSA than FD and controls, and they had higher blood pressure and larger LVEDVi than other groups (p < 0.05). Patients with AL-CA and FD had significant increased E/e′ than HTN patients (Table 1).

**Fig. 1:**
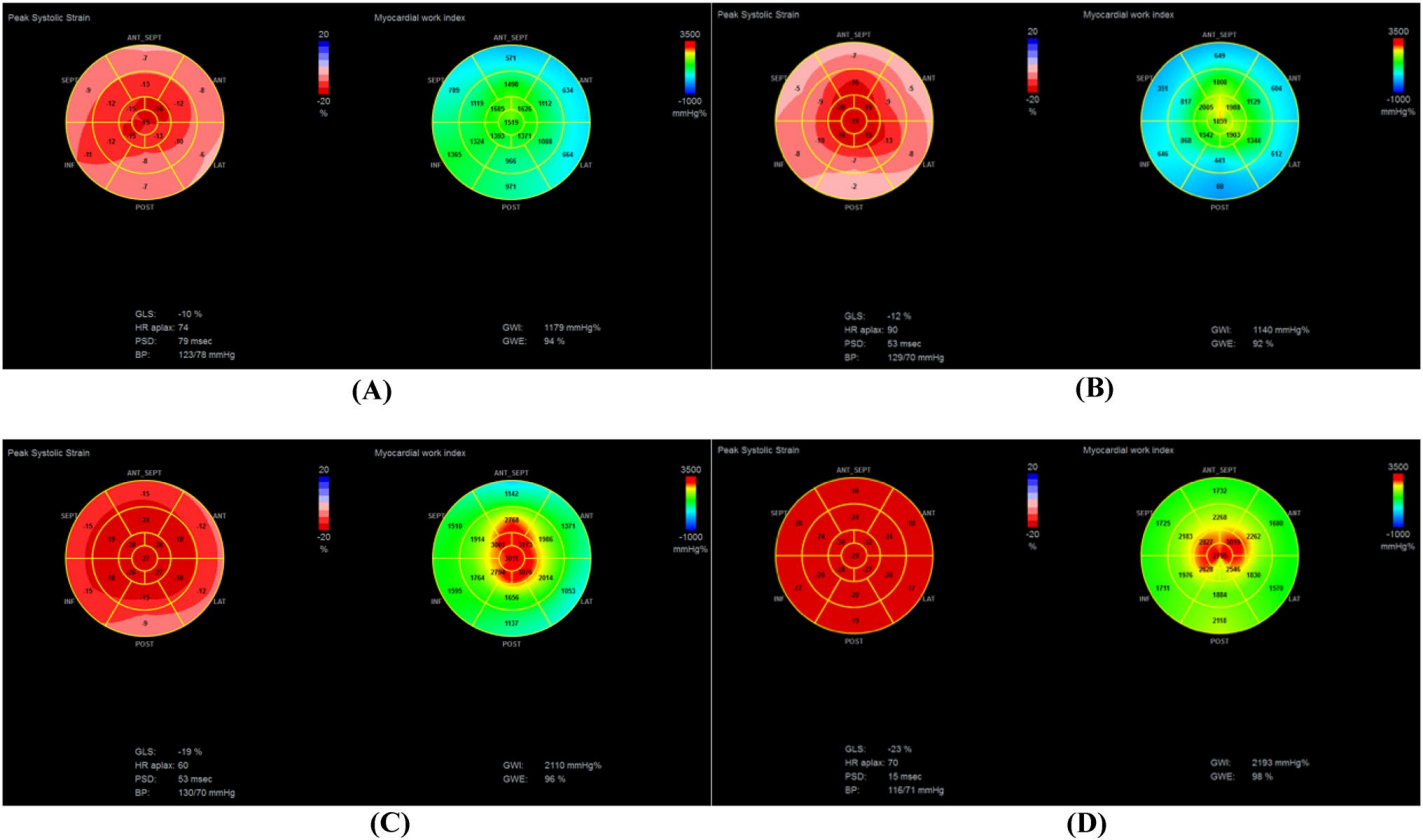
Bull’s eye map for GLS and GWI in patients with left ventricular hypertrophy (all patients had similar interventricular septum thickness of 13 mm) and in healthy control. **A** Fabry disease (GLS: − 10%, GWI: 1179 mmHg%); **B** Cardiac amyloidosis (GLS: − 12%, GWI: 1140 mmHg%); **C** Hypertension (GLS: − 19%, GWI: 2110 mmHg%); **D** Healthy control (GLS: − 23%, GWI: 2193 mmHg%). *GLS* global longitudinal strain, *GWI* global myocardial work index

Compared with healthy controls, patients with FD and AL-CA had reduced GLS, GCW, GWI and GWE and increased GWW and RWT (p < 0.05). However, there was no significant difference in GCW and GWI between HTN patients and controls, although GLS and GWE were significantly decreased in patients with HTN when compared with controls (p < 0.001, p = 0.005 respectively). In patients with FD and AL-CA, GCW, GWI and GWE were more impaired in comparison to HTN patients. When compared to AL-CA patients, FD patients have reduced GLS (p = 0.049), but GWI, GCW, GWW and GWE were similar between AL-CA and FD patients (Table 1). Figure 1 showed bull’s eye map for GLS and GWI in patients with LVH (FD, AL-CA and HTN) (all patients had similar interventricular septum thickness of 13 mm) and in healthy control.

**Table 1:** Demographic, clinical, and echocardiographic characteristics in patients with FD, AL-CA, HTN, and controls

	FD (n = 13)	AL-CA (n = 29)	HTN (n = 72)	Controls (n = 25)
Male (%)	9 (69.23%)	23 (79.31%)	58 (80.56%)	16 (64.00%)
Age (years)	50.00 (38.00, 55.00)	57.00 (52.00, 65.00)^†^	57.00 (42.00, 69.00)^†^	58.00 (41.00, 67.00)
Height (cm)	169.00 (164.00, 175.00)	170.00 (161.00, 172.00)	170.00 (165.00, 175.00)	168.00 (161.00, 171.00)
Weight (kg)	58.50 (54.00, 63.25)	65.00 (60.00, 70.00)	72.00 (65.00, 76.00)*^†^	62.50 (54.00, 72.00)
BSA (m^2^)	1.7 (1.6, 1.8)	1.84 (1.70, 1.90)	1.93 (1.81, 2.00)*^†^	1.78 (1.69, 1.91)
SBP (mmHg)	125.50 (119.50, 139.00)	113.00 (108.00, 121.00)*	145.50 (135.00, 160.00)*^†‡^	124.00 (115.50, 131.00)
DBP (mmHg)	78.13 (11.09)	73.28 (7.01)*	91.15 (14.44)*^†‡^	81.50 (9.24)
LVMI (g/m^2^)	223.89 (189.57, 311.79)*	116.40 (107.13, 138.40)*^†^	113.31 (95.39, 131.35)*^†^	73.96 (67.04, 81.56)
LAVi (ml/m^2^)	42.23 (5.96)*	42.34 (4.06)*	42.22 (4.00)*	34.96 (3.52)
IVSd (mm)	15.00 (13.00, 17.00)*	12.00 (12.00, 13.00)*^†^	12.00 (12.00, 13.00)*^†^	9.00 (8.00, 10.00)
RWT	0.58 (0.52, 0.90)*	0.50 (0.44, 0.54)*^†^	0.42 (0.38, 0.46)^†‡^	0.35 (0.31, 0.38)
LVEDVi (ml/m^2^)	46.08 (5.91)	47.31 (5.21)	50.04 (4.77)*^†‡^	47.48 (3.29)
LVEF (%)	62.00 (57.00, 67.00)	59.00 (56.00, 63.00)*	62.00 (59.00, 65.00)^‡^	64.00 (61.00, 66.00)
E wave (cm/s)	77.00 (70.00, 108.00)*	72.00 (57.00, 92.00)	62.50 (53.50, 73.50)^†‡^	64.00 (58.00, 72.00)
A wave(cm/s)	68.45 (15.98)	79.78 (26.12)	79.01 (21.00)	72.68 (16.30)
E/A	1.40 (1.00, 1.60)*	0.82 (0.73, 1.22)	0.80 (0.60, 1.00)^†^	0.90 (0.80, 1.10)
E/e’	13.56 (12.00, 16.70)*	12.29 (8.35, 18.59)*	9.35 (6.80, 12.15)*^†‡^	6.20 (5.90, 7.50)
TR gradient (mmHg)	26.00 (20.00, 32.00)	25.00 (20.00, 31.00)	23.00 (19.50, 25.00)	21.00 (20.00, 23.00)
GLS (%)	− 12.45 (− 15.25, − 8.50)*	− 15.00 (− 18.65, − 12.00) *^†^	− 20.40 (− 21.95, − 17.25)*^†^	− 22.90 (− 23.60, − 21.10)
GWI (mmHg%)	1312.09 (319.30)*	1387.45 (463.61) *	2273.79 (536.83)^†‡^	2159.52 (253.16)
GCW (mmHg%)	1433.00 (329.78)*	1565.28 (523.40) *	2574.40 (633.59)^†‡^	2457.84 (293.14)
GWW (mmHg%)	93.00 (54.00, 129.00) *	63.00 (46.00, 88.00)*	55.50 (29.50, 76.00)^†^ *	40.00 (20.00, 46.00)
GWE (%)	93.00 (89.00, 95.00)*	93.00 (92.00, 96.00)*	97.00 (96.00, 98.00)*^†‡^	98.00 (97.00, 99.00)
GLS-ABr	1.71 (1.25, 2.29)	1.81 (1.51, 2.30)*	1.58 (1.48, 1.72)*^‡^	1.47 (1.33, 1.57)
GLS-RASp	1.48 (1.24, 1.78)	1.58 (1.35, 1.87) *	1.44 (1.38, 1.55)*	1.34 (1.27, 1.44)
GWI-ABr	1.66 (1.14, 2.30)	1.83 (1.55, 2.39)*	1.57 (1.42, 1.72)*^‡^	1.39 (1.29, 1.52)
GWI- RASp	1.55 (0.56)	1.66 (0.35)*	1.44 (0.21)*^‡^	1.33 (0.10)

*FD* fabry disease, *AL-CA* light chain associated cardiac amyloidosis, *HTN* hypertension, *BSA* body surface area, *SBP* systolic blood pressure, *DBP* diastolic blood pressure, *LVMI* left ventricular mass index, *LAVi* left atrial volume index, *IVSd* interventricular septum dimension, *RWT * relative wall thickness, *LVEDVi* left ventricular end-diastolic volume index, *LVEF* left ventricular ejection fraction, *TR* tricuspid regurgitation, *GLS* global longitudinal strain, *GWI* global work index, *GCW* global constructive work, *GWW* global wasted work, *GWE* global work efficiency, *ABr* apical-to-basal ratio, *RASp* relative apical sparing pattern

*p < 0.05 versus controls; †p < 0.05 versus Fabry disease; ‡ p < 0.05 versus CA

**Fig. 2:**
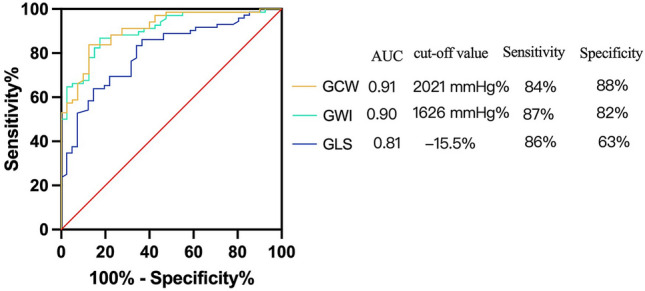
ROC curve for GCW, GWI and GLS discriminating infiltrative cardiomyopathy from HTN. *ROC* receiver operating characteristic, *AUC* area under the curve, *GCW* global constructive work, *GWI* global work index, *GLS* global longitudinal strain

Compared with group of HTN, infiltrative cardiomyopathy (AL-CA and FD) group showed more prominent reduction in GLS, GWI, GCW and GWE, and had significantly larger RWT, E/e’, GWW, GWI-ABr and GWI-RASp (Table 2). Multivariate logistic regression analysis indicated that GWI and GCW could discriminate infiltrative cardiomyopathy from HTN independently after adjustment for BSA, RWT, LVMI, LVEDVi, E/A, E/e’, tricuspid regurgitation (TR) gradient, GWI-ABr and GLS-ABr. Furthermore, GWI and GCW could discriminate between the two groups with high accuracy (AUC 0.90, cut-off value 1626 mmHg%, sensitivity 0.87, specificity 0.82 vs. AUC 0.91, cut-off value 2021 mmHg%, sensitivity 0.84, specificity 0.88) (Fig. 2, Supplementary material Table S2).

**Table 2:** Demographic, clinical, and echocardiographic characteristics in patients with infiltrative cardiomyopathy and hypertension

	Infiltrative cardiomyopathy (n = 42)	HTN (n = 72)	p
Male (%)	32 (76.19%)	58 (80.56%)	0.580
Age (years)	55.00 (49.00, 64.00)	57.00 (42.00, 69.00)	0.510
Height (cm)	170.00 (163.00, 173.00)	170.00 (165.00, 175.00)	0.730
Weight (kg)	62.00 (59.00, 70.00)	72.00 (65.00, 76.00)	< 0.001
BSA (m^2^)	1.79 (1.70, 1.89)	1.93 (1.81, 2.00)	< 0.001
SBP (mmHg)	118.00 (110.00, 123.00)	145.50 (135.00, 160.00)	< 0.001
DBP (mmHg)	74.32 (8.14)	91.15 (14.44)	< 0.001
LVMI (g/m^2^)	134.51 (110.68, 198.79)	113.31 (95.39, 131.35)	< 0.001
LAVi (ml/m2)	41.44 (33.88, 54.11)	39.28 (35.50, 46.42)	0.590
IVSd (mm)	12.50 (12.00, 15.00)	12.00 (12.00, 13.00)	0.031
RWT	0.51 (0.44, 0.62)	0.42 (0.38, 0.46)	< 0.001
LVEDVi (ml/m2)	46.93 (5.39)	50.04 (4.77)	< 0.01
LVEF (%)	60.00 (56.00, 64.00)	62.00 (59.00, 65.00)	0.190
E wave (cm/s)	76.00 (58.00, 94.00)	62.50 (53.50, 73.50)	0.003
A wave (cm/s)	76.50 (23.99)	79.01 (21.00)	0.570
E/A	0.91 (0.78, 1.50)	0.80 (0.60, 1.00)	0.002
E/e′	13.04 (8.59, 17.80)	9.35 (6.80, 12.15)	< 0.01
TR gradient (mmHg)	26.00 (20.00, 32.00)	23.00 (19.50, 25.00)	0.050
GLS (%)	− 14.20 (− 18.06, − 11.00)	− 20.40 (− 21.95, − 17.25)	< 0.001
GWI (mmHg%)	1366.72 (426.16)	2273.79 (536.83)	< 0.001
GCW (mmHg%)	1528.90 (477.64)	2574.40 (633.59)	< 0.001
GWW (mmHg%)	67.50 (46.00, 104.00)	55.50 (29.50, 76.00)	0.023
GWE (%)	93.00 (91.00, 96.00)	97.00 (96.00, 98.00)	< 0.001
GLS-ABr	1.81 (1.44, 2.30)	1.58 (1.48, 1.72)	0.073
GLS-RASp	1.56 (1.33, 1.80)	1.44 (1.38, 1.55)	0.100
GWI-ABr	1.82 (1.44, 2.35)	1.57 (1.42, 1.72)	< 0.001
GWI-RASp	1.63 (0.41)	1.44 (0.21)	< 0.001

*HTN* hypertension, *BSA* body surface area, *SBP * systolic blood pressure, *DBP* diastolic blood pressure, *LVMI* left ventricular mass index, *LAVi* left atrial volume index, *IVSd* interventricular septum dimension, *RWT* relative wall thickness, *LVEDVi* left ventricular end-diastolic volume index, *LVEF* left ventricular ejection fraction, *TR* tricuspid regurgitation, *GLS* global longitudinal strain, *GWI* global work index, *GCW* global constructive work, *GWW* global wasted work, *GWE* global work efficiency, *ABr* apical-to-basal ratio, *RASp* relative apical sparing pattern

**Fig. 3:**
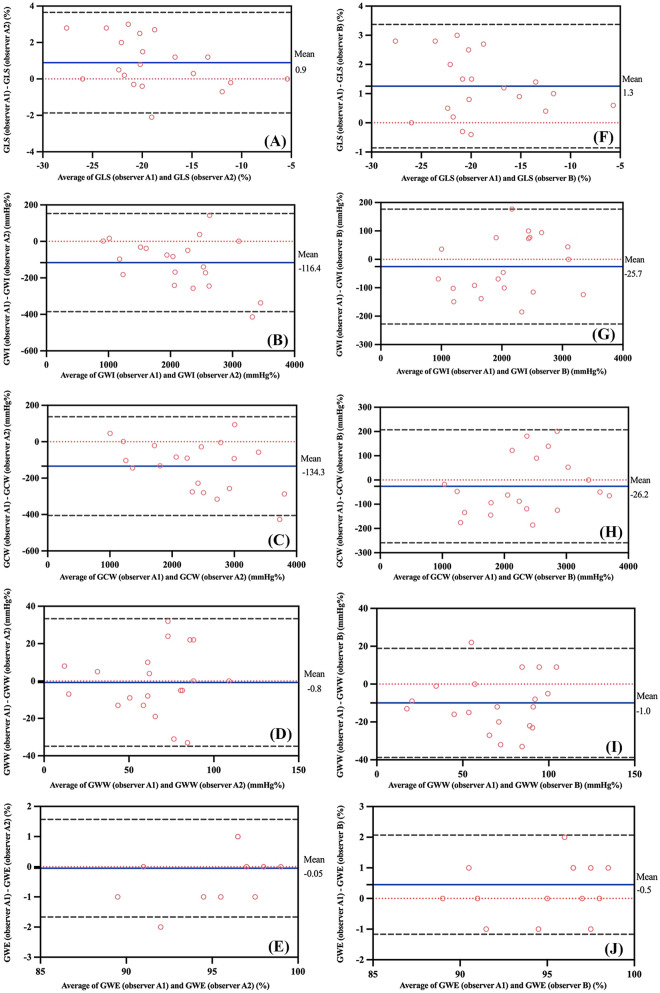
Bland-Altman analysis indicating intra-observer and inter-observer variabilities in GLS and myocardial work indices. **A–E** intra-observer variability; **F–J** inter-observer variability

Excellent intra-observer and inter-observer variabilities were observed for GLS and myocardial work parameters (Fig. 3). Bland-Altman analysis demonstrated low mean differences within the acceptable range.

## Discussion

The current study aimed to demonstrate the value of the novel technique myocardial work for patients with LVH in FD, AL-CA and HTN. The primary findings of this study were: (i) Patients with HTN had reduced GLS and GWE and increased GWW when compared with controls, whereas GWI and GCW were similar between the two groups. (ii) Comparing with healthy control, GLS, GWI, GCW and GWE were reduced and GWW was increased in FD and AL-CA patients, but myocardial work indices could hardly discriminate between FD and AL-CA patients. (iii) GWI and GCW could discriminate infiltrative cardiomyopathy from HTN independently with high accuracy.

In the current study, the FD group had larger LVMI than AL-CA and HTN, it may be explained by the under recognition of Fabry disease in China in the past, which contributes to the patients with FD included in the current study is already in advanced disease progression. The E/e ‘of FD patients was larger than that of other groups, and E/A was greater than 1, indicating a pseudo normal mitral inflow pattern. Therefore, FD patients included in the current study endured more severe left ventricular diastolic dysfunction than AL-CA and HTN patients.

The current study demonstrated that patients with HTN had larger BSA, which is attributed the fact that obesity is one of the risk factors for the attack of hypertension. Patients with HTN had larger LVEDVi, although it was still in normal range, it may result from increased volume overload in hypertension [20].

Previous studies revealed that HTN patients have elevated GWI, GCW and preserved GLS [11, 12], whereas our study showed that hypertension patients had reduced GLS and similar GWI and GCW compared to healthy controls. This could be due to patients in the current study had larger LVMI (median 113.31 g/m^2^), but the LVMI of patients with hypertension in previous studies was smaller (mean 92.3 g/m^2^, median 97 g/m^2^ respectively), which indicates that cardiac involvement was more pronounced in HTN patients in the current study. In early stages of HTN, LV increased the pump function to a higher energy level to compensate the short-term reduction of LV stroke volume. However, chronically increased cardiac loading eventually leads to increased stiffness and LV failure [21, 22]. Hence, we speculate that GWI and GCW increased in the early stage of hypertension and decreased gradually with the progression of the disease.

Fabry disease and AL-CA patients had lower GLS, GWI, GCW, GWE and elevated GWW compared to healthy controls, which is in line with previous studies [9, 23, 24]. FD patients had reduced GLS than patients with AL-CA, but after excluding effects of afterload, there was no statistically significant difference between FD and AL-CA for GWI, GCW and GWE. In the current study FD patients tended to have higher LVMI than patients with AL-CA, but GWI and GCW were similar in the two groups. We speculate that in patients with similar LVMI, those suffering from AL-CA endured more severe left ventricular dysfunction than FD patients, which may be the consequence of light-chain toxicity on cardiac function [25].

The relative apical sparing in left ventricular GLS has been proved as a distinct pattern in CA patients [18], and recently the apical-to-basal segmental work ratio has also been proved having prognostic value by predicting both MACE and all-cause mortality [23]. There is a paucity of studies comparing the relative apical distinct pattern in CA, FD, and hypertension patients. Liu et al. demonstrated that systolic septal longitudinal base-to-apex strain can differ CA patents from FD [26], but the longitudinal strain was measured only in interventricular septal wall, and myocardial work was not measured in their study. Patients with AL-CA had the highest GLS-ABr and GWI-ABr in our study, but there were no statistically significant differences between AL-CA and FD group for the two parameters. Even with limitation of a small sample size, FD patients showed values of both GLS-ABr and GWI-ABr similar to those of AL-CA patients. In FD patients it has been demonstrated a substantial reduction of longitudinal strain in LV basal segments and, with a lesser extent, in middle segments, while LV apex was always preserved [7, 27]. This strain feature cannot be assimilated to the “apical sparing” of cardiac amyloidosis that is more evident and highly sensitive and specific for diagnosis.

The current study showed that patients with infiltrative cardiomyopathy had reduced GLS, GWI, GCW and GWE, and increased E/e’, GWW, GWI-ABr and GWI-RASp compared to HTN. This may be explained by those pathophysiologic mechanisms responsible for LVH in infiltrative cardiomyopathy and hypertension are not the same. Cardiac amyloidosis is associated with the amyloid fibril deposits expand the extracellular space, which results in passive myocardial restriction and dysfunction [1]. Cardiac involvement is a common clinical manifestation in FD patients, chronic intracellular accumulation of glycosphingolipids leads to inflammation, hypertrophy, and interstitial fibrotic process [3]. Both FD and CA are infiltrative cardiomyopathies, progressive deposition of glycosphingolipids or amyloid fibril leads to myocardial dysfunction, reflected as reduced GLS and myocardial work indices in the current study. However, in hypertension, LV enhances pump function against the increased pressure overload [11], so GWI and GCW didn’t reduced significantly in HTN patients within the current study cohort. The current study demonstrated that GWI and GCW could independently differentiate infiltrative cardiomyopathy from HTN with high accuracy. This suggests that myocardial work has an addictive value in differentiating infiltrative cardiomyopathy from hypertension.

There are some limitations to consider in this study. This was a single-center study with potential risks for confounders and biases. Due to the small sample size for FD, the present study does not allow to discriminate between FD and AL-CA, the results need to be confirmed in studies with larger sample size.

## Conclusion

Comparing with healthy controls, GWI and GCW reduced in FD and AL-CA patients, but not in patients with HTN, and all of them had increased GWW and decreased GWE. Myocardial work has an addictive value in differentiating infiltrative cardiomyopathy from HTN.

## Electronic supplementary material

Below is the link to the electronic supplementary material.

Supplementary Material 1

## Data Availability

The datasets used and/or analyzed during the current study are available from the corresponding author on reasonable request.
