# Supplementary material for: The value of myocardial work in patients with left ventricular hypertrophy
Source: Int J Cardiovasc Imaging. 2023 Mar 16;39(6):1105–13. doi: 10.1007/s10554-023-02818-w (PMC10220140; doi:10.1007/s10554-023-02818-w)
Supplement: Supplementary file 1 — Supplementary Material 1 [file 10554_2023_2818_MOESM1_ESM.docx]

Table S1 Genotype and clinical characteristics for FD patients

| No | Age/  gender | Genotype | Classic/Late onset | Extracardiac manifestations | | | | | e-GFR（mL/min/1.73^2^） |
| --- | --- | --- | --- | --- | --- | --- | --- | --- | --- |
|  |  |  |  | Acroparesthesia | Skin | Ears | Renal disease | Brain |  |
| 1 | 51/M | c.395G>A (p.G132E) | Classic | No | No | Yes | Yes | Yes | 80.54 |
| 2 | 40/M | c.827G>T (p.S276I) | Classic | Yes | Yes | Yes | Yes | Yes | 53.07 |
| 3 | 68/F | c.827G>T (p.S276I) | Classic | Yes | No | Yes | Yes | Yes | 5.91 |
| 4 | 41/M | c.298A>T (p.R100X) | Classic | Yes | Yes | Yes | No | Yes | 94.03 |
| 5 | 55/F | c.298A>T (p.R100X) | Classic | Yes | No | Yes | Yes | Yes | 71.34 |
| 6 | 56/F | c.605G>A (p.C202Y) | Classic | Yes | No | No | Yes | No | 64.07 |
| 7 | 36/M | c.1151T>A (p.1384N) | Late onset | No | Yes | No | Yes | No | 109.25 |
| 8 | 38/M | c.708G>A (p. Trp236Ter) and c.709A>T(p.Lys237Ter) | Classic | Yes | Yes | No | Yes | No | 49.80 |
| 9 | 34/F | c.658C>T (p.Arg220Ter) | Late onset | No | No | No | Yes | No | 127.20 |
| 10 | 30/M | c.695T>G（p.Ile232Ser） | Classic | Yes | Yes | No | No | No | 130.30 |
| 11 | 50/M | c.486G>C (p.Trp162Cys) | Late onset | No | No | No | Yes | No | 86.91 |
| 12 | 51/M | c.605G>A (p.Cys202Tyr) | Classic | Yes | Yes | Yes | Yes | Yes | 7.27 |
| 13 | 55/F | c.695T>G（p.Ile232Ser） | Classic | Yes | No | No | Yes | No | 88.33 |

FD: Fabry disease; M: male; F: female

Table S2 Multivariate logistic regression analysis for GLS and myocardial work indices discriminating infiltrative cardiomyopathy from HTN.

|  | |  | OR | CI95% |  | p | AUC |
| --- | --- | --- | --- | --- | --- | --- | --- |
| GWI | GWI | | 0.63 | 0.49 | 0.82 | <0.001 | 0.96 |
|  | BSA | | 0.02 | 0 | 8.37 | 0.202 |  |
|  | LVMI | | 1.00 | 0.98 | 1.01 | 0.600 |  |
|  | RWT | | 1.05 | 0.93 | 1.19 | 0.443 |  |
|  | LVEDVi | | 0.94 | 0.88 | 1.01 | 0.07 |  |
|  | E/A | | 5.36 | 0.51 | 56.30 | 0.162 |  |
|  | E/e' | | 1.03 | 0.80 | 1.31 | 0.839 |  |
|  | TR gradient | | 1.13 | 0.96 | 1.33 | 0.139 |  |
|  | GWI-ABr | | 1.80 | 0.06 | 56.50 | 0.737 |  |
|  | GLS-ABr | | 0.96 | 0.03 | 28.57 | 0.980 |  |
| GCW | GCW | | 0.67 | 0.53 | 0.84 | 0.001 | 0.95 |
|  | BSA | | 0.02 | 0 | 6.99 | 0.187 |  |
|  | LVMI | | 0.99 | 0.97 | 1.01 | 0.535 |  |
|  | RWT | | 1.04 | 0.93 | 1.15 | 0.517 |  |
|  | LVEDVi | | 0.94 | 0.88 | 1.00 | 0.083 |  |
|  | E/A | | 5.01 | 0.48 | 52.20 | 0.178 |  |
|  | E/e' | | 1.04 | 0.83 | 1.31 | 0.74 |  |
|  | TR gradient | | 1.10 | 0.95 | 1.29 | 0.199 |  |
|  | GWI-ABr | | 1.86 | 0.09 | 39.70 | 0.693 |  |
|  | GLS-ABr | | 0.91 | 0.05 | 18.07 | 0.948 |  |
| GLS | GLS | | 0.81 | 0.66 | 1.1 | 0.058 | 0.90 |
|  | BSA | | 0.02 | 0 | 2.46 | 0.110 |  |
|  | LVMI | | 1.00 | 0.99 | 1.02 | 0.652 |  |
|  | RWT | | 1.03 | 0.94 | 1.12 | 0.507 |  |
|  | LVEDVi | | 0.95 | 0.90 | 1.01 | 0.121 |  |
|  | E/A | | 3.81 | 0.50 | 29.28 | 0.199 |  |
|  | E/e' | | 1.05 | 0.87 | 1.27 | 0.622 |  |
|  | TR gradient | | 1.10 | 0.97 | 1.23 | 0.130 |  |
|  | GWI-ABr | | 5.79 | 0.35 | 94.71 | 0.218 |  |
|  | GLS-ABr | | 0.58 | 0.04 | 8.52 | 0.695 |  |
| GWE | GWE | | 0.81 | 0.64 | 1.01 | 0.063 | 0.89 |
|  | BSA | | 0.05 | 0 | 6.34 | 0.220 |  |
|  | LVMI | | 1.01 | 0.99 | 1.03 | 0.364 |  |
|  | RWT | | 1.05 | 0.96 | 1.15 | 0.293 |  |
|  | LVEDVi | | 0.94 | 0.88 | 1.00 | 0.060 |  |
|  | E/A | | 4.91 | 0.68 | 35.41 | 0.115 |  |
|  | E/e' | | 1.06 | 0.87 | 1.29 | 0.554 |  |
|  | TR gradient | | 1.09 | 0.96 | 1.23 | 0.186 |  |
|  | GWI-ABr | | 3.20 | 0.18 | 56.19 | 0.426 |  |
|  | GLS-ABr | | 0.93 | 0.06 | 14.49 | 0.957 |  |
| GWW | GWW | | 1.00 | 0.98 | 1.02 | 0.874 | 0.89 |
|  | BSA | | 0.02 | 0 | 2.64 | 0.114 |  |
|  | LVMI | | 1.01 | 1.00 | 1.03 | 0.127 |  |
|  | RWT | | 1.07 | 0.98 | 1.17 | 0.123 |  |
|  | LVEDVi | | 0.96 | 0.90 | 1.02 | 0.183 |  |
|  | E/A | | 3.75 | 0.53 | 26.43 | 0.185 |  |
|  | E/e' | | 1.09 | 0.90 | 1.30 | 0.374 |  |
|  | TR gradient | | 1.06 | 0.94 | 1.19 | 0.288 |  |
|  | GWI-ABr | | 6.18 | 0.40 | 94.40 | 0.190 |  |
|  | GLS-ABr | | 0.57 | 0.04 | 8.17 | 0.678 |  |

HTN: hypertension; GWI: global work index; BSA: body surface area; LVMI: left ventricular mass index; RWT: relative wall thickness; LVEDVi: left ventricular end-diastolic volume index; TR: tricuspid regurgitation; ABr: apical-to-basal ratio; GLS: global longitudinal strain; GCW: global constructive work; GWE: global work efficiency; GWW: global wasted work; AUC: area under the curve.
